# Implementation of Intergenerational Poetic Portraiture and Virtual Reality Programming as Companion Activities

**DOI:** 10.1093/geroni/igaf122.1589

**Published:** 2025-12-31

**Authors:** Kelly Munly, Erin Murphy, Nick Smerker

**Affiliations:** Penn State Altoona, Altoona, Pennsylvania, United States; The Pennsylvania State University, University Park, Pennsylvania, United States; The Pennsylvania State University, University Park, Pennsylvania, United States

## Abstract

Human Development and Family Studies (HDFS) and English faculty at a small campus collaborated to support advanced students of Adulthood Across the Lifespan and Social Perspectives of Aging (N = 28) to be trained in both poetic portraiture and application of virtual reality tools to support exploration of the life course context of older adults they prepared to work with in a long-term care practicum experience. This effort to prepare students for intergenerational programming is part of a larger effort toward Age Friendliness at this campus and in the surrounding community. Life course theory is the foundation of project aims and design. After HDFS faculty established a plan for collaboration with the English program, English faculty provided students with training in poetic portraiture as both a potential qualitative research method and as a foundation for conversations with older adults served in the practicum. Subsequently, Media Commons trainers provided classes with training in using virtual reality headsets to support older adults’ opportunity to reengage with prior experiences (e.g., farm life or travel) and to take on exploration of new travel destinations, adding to life course experience. The poetic portraiture activity provided foundational conversations and insights into lived experiences, informing the subsequent explorations through virtual reality. Students followed their practicum training and experiences with reflections and forum discussions on their Canvas course online platform, with opportunities to share and analyze what they learned in the context of life course understanding.

